# A non-stop S-antigen gene mutation is associated with late onset hereditary retinal degeneration in dogs

**Published:** 2013-08-27

**Authors:** Orly Goldstein, Julie Ann Jordan, Gustavo D. Aguirre, Gregory M. Acland

**Affiliations:** 1 Baker Institute for Animal Health, Cornell University College of Veterinary Medicine, Ithaca, NY; 2 School of Veterinary Medicine, University of Pennsylvania, Philadelphia, PA

## Abstract

**Purpose:**

To identify the causative mutation of canine progressive retinal atrophy (PRA) segregating as an adult onset autosomal recessive disorder in the Basenji breed of dog.

**Methods:**

Basenji dogs were ascertained for the PRA phenotype by clinical ophthalmoscopic examination. Blood samples from six affected cases and three nonaffected controls were collected, and DNA extraction was used for a genome-wide association study using the canine HD Illumina single nucleotide polymorphism (SNP) array and PLINK. Positional candidate genes identified within the peak association signal region were evaluated.

**Results:**

The highest -Log_10_(P) value of 4.65 was obtained for 12 single nucleotide polymorphisms on three chromosomes. Homozygosity and linkage disequilibrium analyses favored one chromosome, CFA25, and screening of the S-antigen (*SAG*) gene identified a non-stop mutation (c.1216T>C), which would result in the addition of 25 amino acids (p.*405Rext*25).

**Conclusions:**

Identification of this non-stop *SAG* mutation in dogs affected with retinal degeneration establishes this canine disease as orthologous to Oguchi disease and *SAG*-associated retinitis pigmentosa in humans, and offers opportunities for genetic therapeutic intervention.

## Introduction

The gene S-antigen (*SAG*), also known as arrestin, encodes a major soluble rod outer segment protein that exemplifies a family of inhibitory proteins that bind tyrosine-phosphorylated receptors to block their interaction with specific G-proteins to terminate a signal chain. In the retina, arrestin binds to activated rhodopsin [1] in rod outer segments, to hinder G-protein binding and quench rhodopsin activity [2,3]. Mutations in this gene in humans have been associated with Oguchi disease, a rare form of autosomal recessive stationary night blindness (HGMD). This disease has an unusual but characteristic clinical feature, the Mizuo-Nakamura phenomenon, in which an unusual golden-yellow discoloration of the fundus disappears in the dark-adapted condition and reappears shortly after exposure to light [4]. Patients with Oguchi disease usually have night blindness but normal color vision and normal cone function [5,6]. Patients exhibiting Oguchi disease may later develop retinitis pigmentosa (RP), and in other cases, the presence of RP can mask signs of Oguchi disease [7-12].

Progressive retinal atrophy (PRA) comprises a group of genetically inherited diseases affecting dogs of various breeds. Similar to RP in humans, PRA is characterized by photoreceptor degeneration causing progressive vision loss, culminating in blindness. This is a highly heterogeneous group of diseases with more than 12 different causative gene mutations already identified in canine populations, causing either early or late onset disease, and inherited as autosomal dominant, autosomal recessive, or X-linked [for a review, see 13]. These canine mutations result in phenotypes resembling orthologous human diseases and provide valuable large animal models for studying the molecular basis of the diseases, and for preclinical trials of potential therapies.

In the Basenji breed, the adult onset of PRA was observed, with initial visual loss in dim light (night blindness), which gradually progressed to total blindness. Initial visual loss affects the peripheral visual field, but unless the dog is used for high visual performance tasks such as agility work, the reduction in the visual field (tunnel vision) may not be apparent. Despite tunnel vision and night blindness, many Basenjis affected with PRA retain adequate forward daylight vision for many years, sometimes for their entire natural life. This phenotype highly resembles progressive rod-cone degeneration (*prcd*), late onset retinal degeneration affecting multiple breeds caused by a point mutation in the *PRCD* gene [14]. A complementary breeding test for dogs affected with *prcd* excluded allelism between these two similar diseases [15], and this has been confirmed with *PRCD* genotyping. Complicating this broadly recognizable clinical phenotype has been considerable heterogeneity in the disease manifestation, and uncertainty about whether this clinical heterogeneity represented a single protean disorder or an underlying genetic heterogeneity. This problem has been further complicated by the extremely small gene pool of Basenjis in the American breeding population and the multiple inbreeding loops apparent in pedigree analysis.

Linkage disequilibrium (LD) enables genome-wide association mapping in populations even when the extended pedigree information normally required for linkage analysis is either incomplete or missing entirely. Furthermore, the development of genome-wide single nucleotide polymorphism (SNP) chip arrays has made such mapping widely available for humans and several other species, including the dog. In humans, mapping has been particularly successful in isolated populations [16-20]. In the broader canine population, each breed comprises such a genetically isolated population, with extensive LD blocks, making the association approach attractive for mapping traits and disease-causative genes [21-25].

In this study, we report a genome-wide association study (GWAS) to map a specific form of PRA in the Basenji breed of dog, using six affected dogs and three control dogs. Together with homozygosity analysis and consideration of the mode of inheritance, we identified a non-stop mutation in *SAG* that cosegregated with the disease phenotype and caused the disease. This creates an opportunity to study the molecular basis of *SAG*-associated retinal diseases, and a model for evaluating potential therapeutic approaches for Oguchi disease and *SAG*-associated RP in humans.

## Methods

### Samples

All procedures involving animals were conducted following the guidelines of the Institute for Laboratory Animal Research (Guide for the Care and Use of Laboratory Animals), the U.S. Public Health Service (Public Health Service Policy on Humane Care and Use of Laboratory Animals), and the Association for Research in Vision and Ophthalmology (ARVO) Statement for the Use of Animals in Ophthalmic and Vision Research. Blood samples were collected by cephalic or jugular venepuncture into vacutainer tubes containing ethylenediaminetetraacetic acid (EDTA) anticoagulant from 80 purebred Basenji dogs: 19 diagnosed clinically as affected with PRA, six diagnosed clinically as *tentatively* affected with PRA, 18 nonaffected controls beyond the assumed age of risk (at least 6 years of age), and 37 with no assigned phenotype either because they were younger than the assumed age of risk or due to lack of information. Blood was stored at 4 °C or -20 °C until DNA extraction. DNA was similarly extracted from blood samples of 110 purebred dogs from 22 different breeds not known to segregate this disease (Appendix 1).

### Phenotypic evaluation of study dogs

Ascertainment of disease phenotype was based entirely on indirect ophthalmoscopic examination by board-certified veterinary ophthalmologists. In the minority of cases, the ophthalmoscopic diagnosis was confirmed with clinical electroretinography. The cases studied in the present report include only dogs examined and diagnosed by either or both of two of the authors (GMA, GDA), although in several cases the initial diagnostic examination was previously performed by other veterinarians.

### Genome-wide association study

Of the 19 purebred Basenji dogs diagnosed clinically as affected with PRA, six were selected as cases for association analysis, based on confidence in the disease ascertainment (dogs affected with stage II PRA), consistency of the disease phenotype among the selected dogs, pedigree information supporting autosomal recessive inheritance identical by descent (IBD), and choosing the least closely related set of dogs affected with PRA available (four dogs had no parents or grandparents in common).

Among 43 Basenji dogs not expressing PRA symptoms, three dogs not affected with PRA were selected for the control group based on having no parents or grandparents in common with each other, being clinically diagnosed as nonaffected with PRA at an age beyond the apparent age of risk for onset of the disease (ages 11.7, 7.5, and 6 years old), being free of other ocular abnormalities that might obscure or confuse the presence of PRA, and being close relatives of the affected dogs chosen as cases. The unaffected dog at age 6, although borderline for the age of onset, was a sibling of an affected dog, and was chosen to accomplish the fourth criterion.

Samples were genotyped using the Illumina Canine SNP chip (HD Canine SNP Chip), which comprises 173,662 SNP loci, following the manufacturer's standard protocol. Genotypes were called using GenomeStudio (Illumina, San Diego, CA). Genotype calls were converted into Plink-format files, and association was tested using the association command without pedigree or sex information (PLINK) [26]. The genotype calls for chromosomes 4, 13, and 25 were retrieved from the files and assembled into haplotypes to identify homozygous blocks.

### Homozygous block analysis

Homozygous block analysis was performed on affected dogs only (n=6) using Plink, under the following criteria: sliding window criteria: 1,000 Kb, 150 SNPs, five missing calls, one heterozygous call, 0.05 threshold; homozygous segment criteria: length 1,000 Kb, 150 SNPs, 50 density (Kb/SNP). The output was then filtered for those chromosomes where all six cases showed a minimum of one homozygous segment anywhere in the chromosome. The segments then were aligned within each chromosome, to identify those where all six dogs shared the same homozygous block. For all such regions, genotype calls were retrieved to determine whether all homozygous blocks were homozygous for the same haplotype. If so, then the “affected haplotype” was compared to haplotypes observed in the control group.

### Candidate gene analysis

Within a homozygous block on chromosome 25 (CFA25), two genes were considered candidates: S-antigen, arrestin (*SAG*), which has been associated with Oguchi disease, as well as retinitis pigmentosa [27-29], and potassium inwardly-rectifying channel, subfamily J, member 13 (*KCNJ13*), which has been associated with snowflake vitreoretinal degeneration and Leber congenital amaurosis [30,31].

*SAG* screening was performed on five dogs: one Basenji affected with PRA and its nonaffected litter-mate, two nonaffected Basenji dogs, and one normal Boxer dog. Eighteen primer pairs were used to amplify the 16 coding exons, as well as two intronic regions rich with single nucleotide polymorphisms (Appendix 2: A). *KCNJ13* gene screening was conducted on six dogs: two affected Basenji dogs, three unaffected Basenjis, and one normal Boxer dog. Nine primer pairs were designed to amplify the three exons of the gene (Appendix 2B). PCR products of both genes were sequenced, and sequences aligned using Sequencher 4.2.2 Software (Gene Codes, Ann Arbor, MI).

### S-antigen mutation screening in the Basenji population and in other breeds

The identified *SAG* non-stop mutation was genotyped on the complete set of Basenji dogs available (N=80) with PCR using primer pairs flanking the mutation (Appendix 2A, pair number 18), followed by sequencing. An allele-specific extension test was then designed to screen 110 dogs from 22 breeds (Appendix 1). One primer pair, containing a forward primer specific to the wild-type allele, amplifies a 259 bp fragment, and a separate primer pair, containing a forward primer specific to the mutant allele, amplifies a 531 bp product (Appendix 2C). Both forward primers contain a mismatch in the 3′ penultimate base.

## Results

### Clinical evaluation

PRA as a clinical disease in the Basenji was, in broad terms, typical adult onset canine retinal degeneration. The first ophthalmoscopically observed evidence of disease was irregular hypo- and hyperreflectivity of the tapetal fundus. This phenomenon, taken as evidence of retinal thinning, is referred to as stage I or early stage PRA*.* Basenjis affected with PRA typically exhibited evidence of stage I PRA at about 5 years of age. These dogs when reexamined ophthalmoscopically at a later age typically showed progression of disease by thinning (attenuation) of the retinal vasculature, which usually became ophthalmoscopically evident by 6 or 7 years of age. This phenomenon, taken as evidence of reduced blood flow through the retinal vasculature, is referred to as stage II or mid-stage PRA*.* In all of these respects, Basenjis affected by PRA closely resemble dogs of other breeds affected by the *prcd* form of PRA.

Clinical diagnosis of PRA in the Basenji was complicated by several factors, however. These included a common prevalence of conus in dogs affected with PRA and nonaffected dogs. Conus refers to a roughly triangular region immediately superior to the optic nerve head in which the tapetum is yellow (and may be hyperreflective) compared to the surrounding green tapetal region. This phenomenon is seen, though less frequently, in normal dogs of other breeds, but also is seen in dogs of other breeds as one of the earliest signs of retinal thinning. In the fundus of Basenjis affected with PRA, this area of conus is sometimes observed to expand and become hyperreflective. Second, the optic nerve head is typically less myelinated and thus less extensive over the fundus than is typical of other breeds of dog. Also seen repeatedly in Basenjis affected with PRA and nonaffected Basenjis was a strange mottled golden-yellow-brown discoloration of the tapetal fundus that obscured the tapetal reflection in a patchy manner, and gave the fundus a beaten bronze appearance. This fundus change, termed bronzing, could either mask the earliest signs of retinal degeneration in some Basenjis affected with PRA or produce a mottled variation in tapetal reflectivity that to some extent mimicked the earliest signs of retinal degeneration in Basenjis that eventually proved to be not affected with PRA.

**Figure 1:**
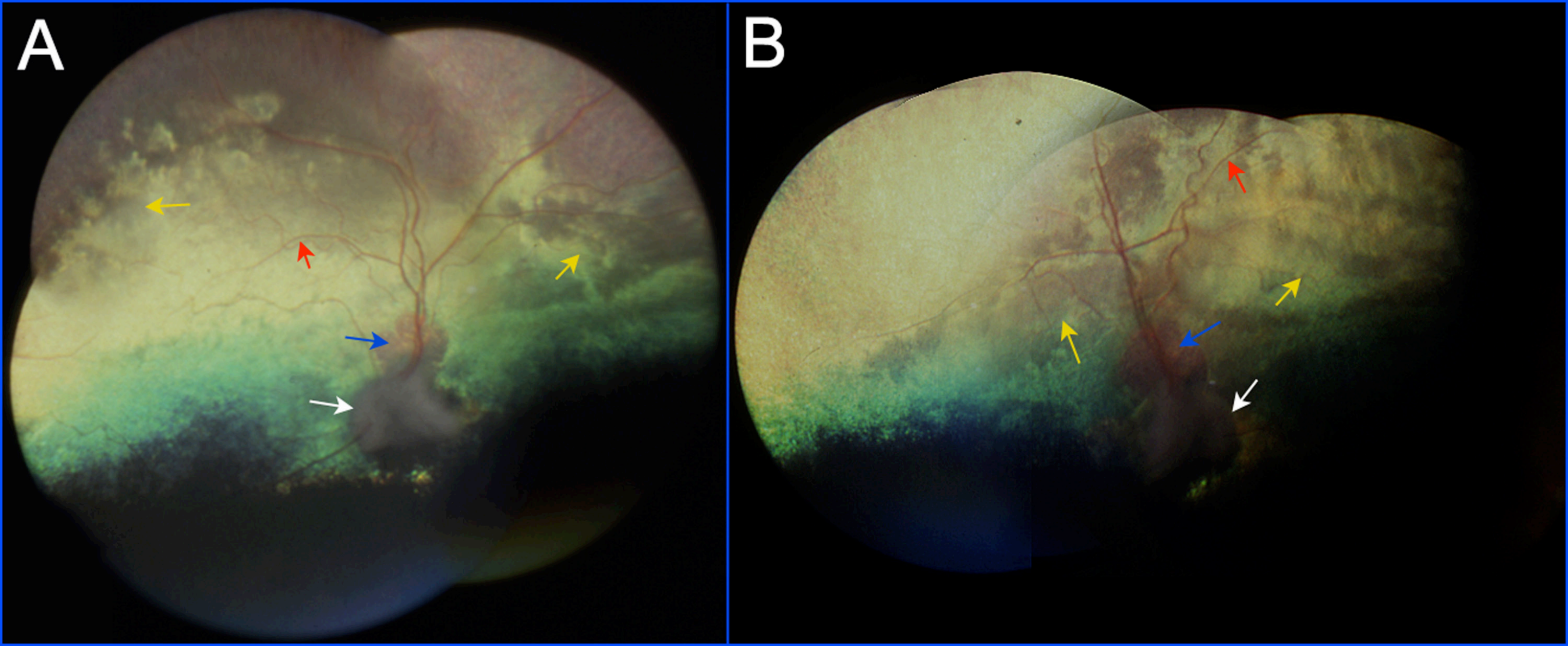
Fundus photographs of a nine-year-old Basenji dog affected with progressive retinal atrophy (PRA; Dog 6, Figure 2). **A**: Fundus photographs of the right eye were assembled into a montage. **B**: Fundus photographs of the left eye were assembled into a montage. Retinal degeneration is evidenced by the thin retinal blood vessels (red arrows), pallid optic nerve head (white arrows), and an irregular pattern of tapetal reflectivity (yellow arrows). The yellow region immediately superior to the optic nerve head in both eyes (blue arrows) is an expanded area of conus (see text).

Finally, the fundus appearance in Basenjis affected with PRA was often patchy, or non-uniform, within a given eye, between the two eyes of a given affected dog, and among affected dogs. To some extent, this fundus appearance is observed in other forms of canine PRA, but was particularly evident in the eyes of Basenjis affected with PRA (Figure 1).

### Pedigree analysis and selection of cases and controls

**Figure 2:**
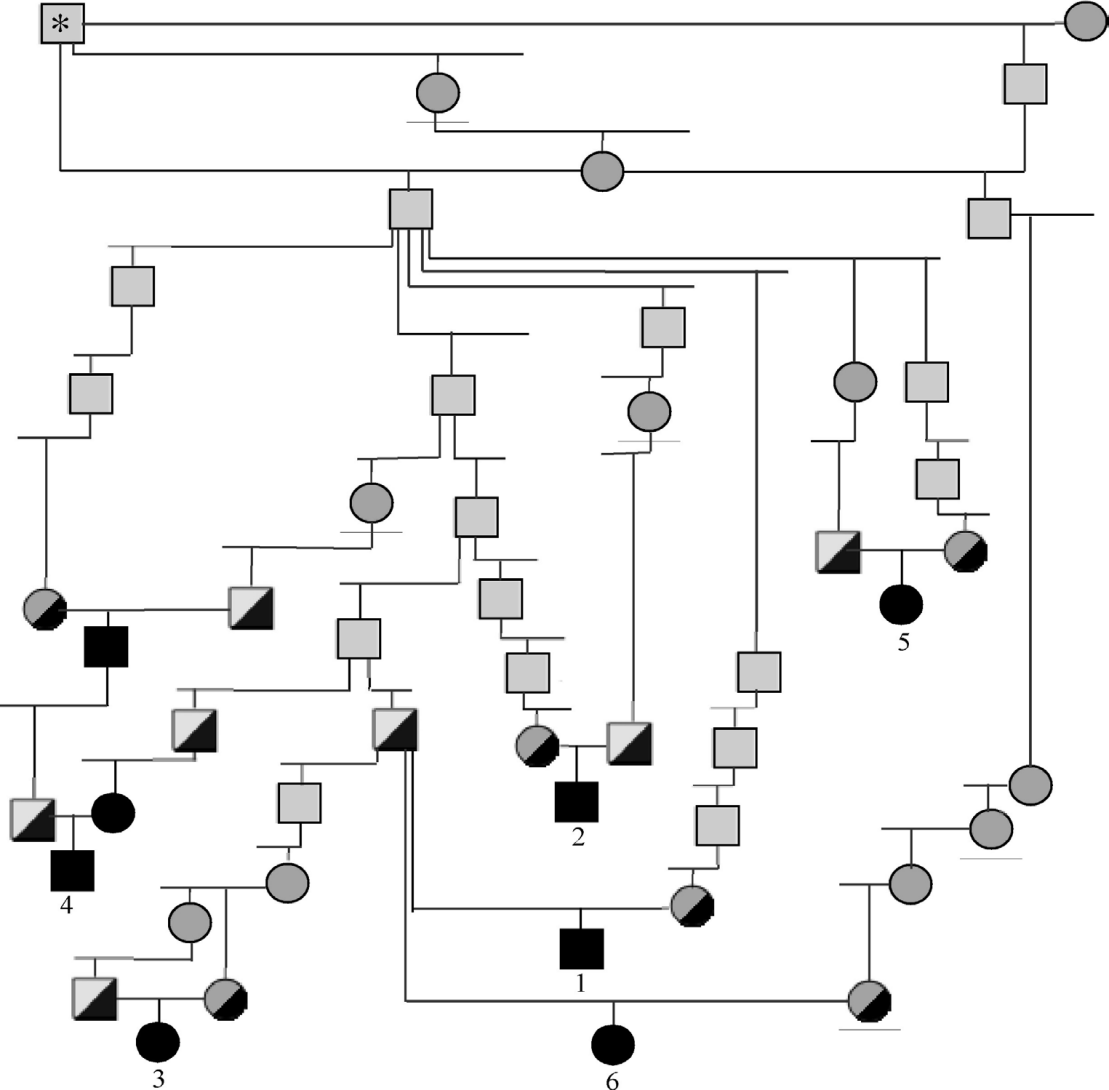
Pedigree of the six Basenji dogs affected with progressive retinal atrophy (PRA) selected as cases for the genome-wide association study. Square symbols=males; Circles=females. Solid black symbols=affected dogs; Grey symbols=dogs of unknown phenotype; half-black/half-gray symbols=dogs that, assuming autosomal recessive inheritance, are at least obligate heterozygotes. The male dog marked with an asterisk is common to the maternal and paternal ancestral lines of all six affected dogs in this pedigree. Not all ancestors or lines of descent are shown.

The breeding population of Basenjis in the United States of America (i.e., Basenjis registered with the American Kennel Club) descends from a small number of founders, and has a complicated pedigree structure with multiple inbreeding loops and overlapping generations. In combination with the late age of onset, and the heterogeneous clinical nature of the disease, this made pedigree analysis more uncertain than usual in terms of establishing the likely mode of inheritance. A subset of six affected dogs were thus chosen for the genome-wide association study with criteria (see the Methods) intended to maximize the likelihood that all six dogs were affected with a single autosomal recessive disease inherited from a common ancestor, and thus identical by descent (Figure 2).

### Genome-wide association study results

**Figure 3:**
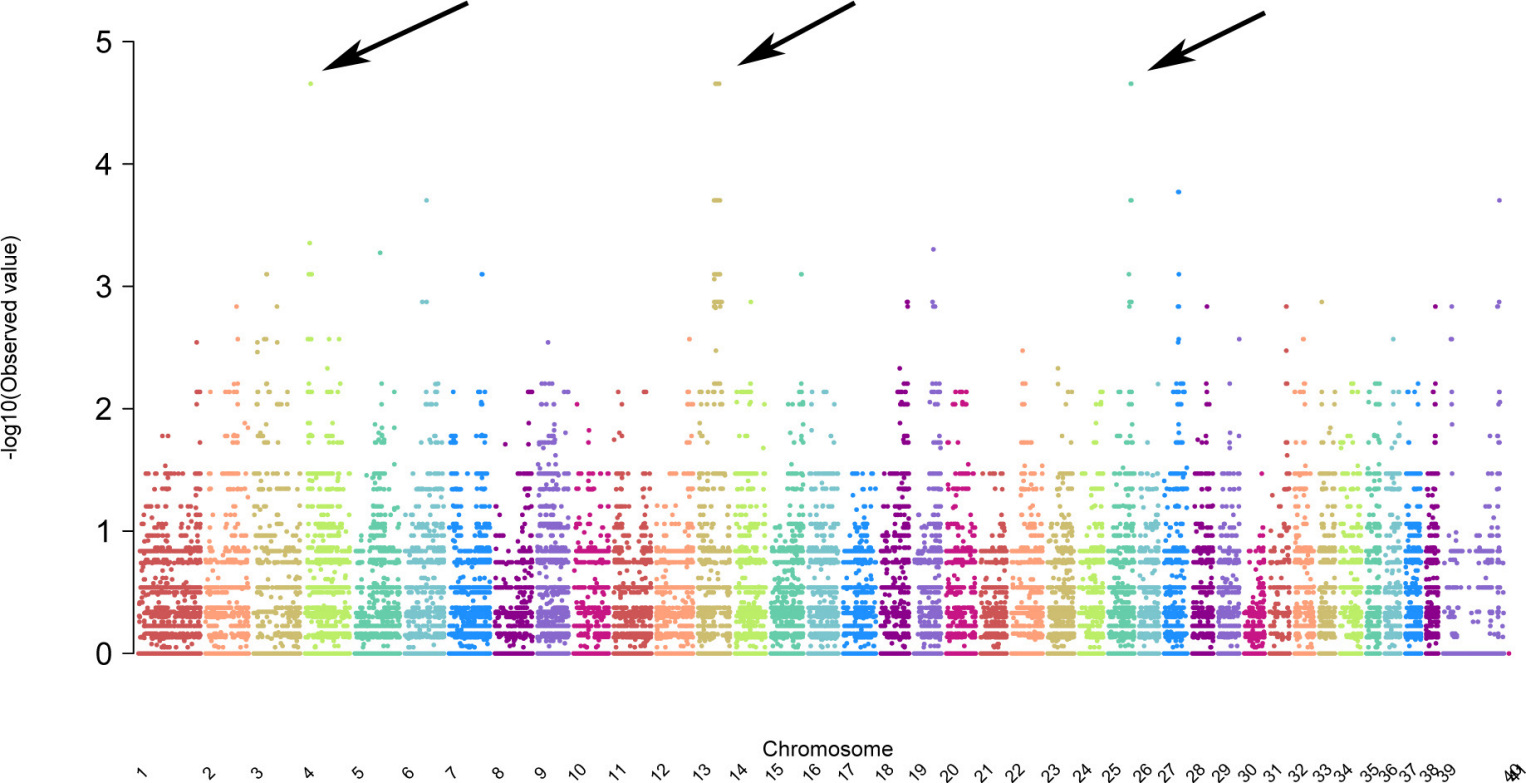
Manhattan plot presenting genome-wide association study results in Basenji progressive retinal atrophy (PRA). y-axis=Probability statistic (–log_10_(P)) for association test analyzed using PLINK. X-Axis=single nucleotide polymorphisms ordered on chromosomes (chromosomes 39 and 41 represent the X-chromosome, and chromosome 40 the Y chromosome). The highest –log_10_(P) values are observed on CFA4, CFA13, and CFA25 as denoted by the black arrows.

Nine dogs were genotyped for the 173,662 SNPs on the HD Canine Illumina array: six affected with PRA and three controls. Genotype calls were converted to Plink files, and association analysis was run comparing the six affected dogs to the three controls, without considering pedigree relationship or gender. The highest -Log_10_(P) value of 4.65 was obtained for 12 SNPs: one SNP on CFA4, seven on CFA13, and four SNPs on CFA25 (Table 1; Figure 3). All affected dogs were homozygous for the same allele of the single CFA4 SNP (at position 14,140,022), and all non-affected dogs were homozygous for the complementary allele. Haplotype analysis in a 2 Mb interval surrounding this SNP (13,146,696–15,141,399), identified a 1.05Mb block of homozygosity in the control group (13,378,063–14,426,721), and heterozygosity in five out of the six affected dogs (data not shown). Since the cross-breeding test with dogs affected with PRCD suggested that the disease is recessive [15], this locus was not further investigated.

**Table 1:** Peak Genome-wide Association Results.

#	**Chromosome**	**SNP**	**Base position**	**Size (Mb) of homozygous block observed in affected dogs and location (bp)**
1	4	BICF2P1179952	14,140,022	Not observed
2	13	BICF2P1417808	37,566,130	0.16 (37,480,062–37,641,454)
3		BICF2S23534826	38,102,290	0.24 (38,057,700–38,300,049)
4		BICF2P802958	38,149,473	
5		BICF2G630659956	39,187,805	0.93 (39,172,713–40,101,896)
6		BICF2G630659916	39,220,108	
7		BICF2S23222210	42,456,241	0.045 (42,415,530–42,460,613)
8		BICF2P1192547	44,870,466	0.17 (44,818,724–44,987,699)
9	25	BICF2G630105512	47,523,118	2.09 (46,893,645–48,980,899)
10		BICF2P320510	47,531,764	
11		BICF2P1326525	48,454,294	
12		BICF2P1326531	48,455,885	

Genome-wide association analysis of CanineHD Illumina SNP array data for 6 PRA-affected Basenji dogs and 3 controls, identified 12 SNPs sharing the highest -log_10_P-value of 4.6558. The SNP in CFA4 was not within a homozygous block. Seven SNPs in CFA13 were within three different homozygous blocks, each smaller than 1 Mb. Four SNPs in CFA25 were within a single 2.09 Mb homozygous block.

Affected dogs were all homozygous for one allele at all seven SNPs on CFA13, and nonaffected dogs were homozygous for the complementary allele. Haplotype analysis of the approximately 7 Mb interval (37,566,130–44,870,466) spanning these seven SNPs in affected dogs showed five homozygous blocks containing one or two of these seven significant SNPs, with distances ranging from 45 Kb to 929 Kb (Table 1). Although, assuming recessive inheritance, each block qualified as a candidate region, we anticipated from consideration of the genomic structure of the Basenji breed that the LD block carrying the mutation for the PRA disease would be larger than 1 Mb [32]. Furthermore, examination of the 0.9 Mb homozygosity interval on CFA13 (39,172,713–40,101,896) in the CanFam2 genome assembly did not reveal any genes appealing as candidates for a retinal degenerative disorder.

The four SNPs on CFA25 were all within one 2.09 Mb homozygosity block (46,893,645–48,980,899), in which all affected dogs were homozygous for one allele for each SNP, and the three unaffected dogs were homozygous for the complementary allele. One SNP at position 48,147,815 appeared to break this homozygosity block into two blocks of 1.25 Mb and 0.83 Mb, but this was considered unreliable as all genotype calls for this SNP were either heterozygous or no call (Appendix 3).

### Homozygosity analysis

Genotype calls from the affected dogs only (n=6) were analyzed for homozygosity blocks greater than 1.0 Mb, excluding sex chromosomes. Five hundred and thirty-nine such blocks were identified. After sorting by chromosome and aligning homozygous regions within each chromosome, five loci were identified where all six dogs shared a homozygous segment (Table 2): CFA 6, 17, 21, 25, and 35 with shared block sizes of 2.94, 2.96, 2.73, 1.88, and 0.78 Mb, respectively. Haplotype analysis confirmed that all six affected dogs were homozygous for the same haplotype at each region and reduced the sizes of the homozygous blocks to 2.2, 3.1, 2.84, 2.09, and 0.73 Mb, respectively (Table 2). Two of the three control dogs shared the same genotype as the affected dogs in all loci except CFA25. None of the three control dogs carried the risk haplotype on CFA25.

**Table 2:** Homozygosity analysis, CanineHD Illumina SNP array data in 9 Basenji dogs, 6 cases and 3 controls.

**#**	**CFA**	**Start position**	**End position**	**Size in Mb**	**Homozygous block location (bp) and size (Mb) after haplotype analysis**	**Inclusion of locus after comparing to controls**
1.	6	3,018,918	5,962,902	2.94	3,692,623- 5,896,276 (2.2)	No
2.	17	19,570,751	22,531,631	2.96	19,570,699- 22,673,502 (3.1)	No
3.	21	5,144,127	7,870,758	2.73	5,095,371- 7,937,895 (2.84)	No
4.	25	47,030,007	48,906,876	1.88	46,893,645- 48,980,899 (2.09)	**Yes**
5.	35	14,038,185	14,818,637	0.78	14,091,098- 14,818,637 (0.73)	No
**Total**	**5 Loci**			**11.29 Mb**		

Five homozygous blocks were identified with sizes between 0.78 and 2.96 Mb. The homozygous block on CFA25 was exclusively present in the 6 PRA-affected dogs (cases), all other blocks were observed in both cases and controls.

### Evaluation of candidate genes

From association and homozygosity analyses, the association peak on chromosome 25 was considered the best potential candidate region to investigate further. Within the 2.09 Mb homozygosity block encompassing all four SNPs with the maximum p value, two genes were evaluated as positional candidates: *KCNJ13* and *SAG*. Primer pairs were designed to amplify all exons of both genes, including flanking sequences from the 5′ untranslated region (UTR), introns, and 3′ UTR (Appendix 2A, B), and used to generate amplicons from DNA samples representing six dogs: two affected Basenji dogs, three non-affected Basenjis, and one normal Boxer dog.

*KCNJ13*, previously associated with snowflake vitreoretinal degeneration and Leber congenital amaurosis in humans [30,31], was evaluated with sequence analysis of nine amplicons (Appendix 2B) encompassing all three exons. Only one polymorphism was identified (exon 2), and was not in association with the disease (Appendix 4: A). *KCNJ13* was not further investigated as a candidate.

*SAG*, as identified in the Canfam2 genome sequence at chr25:47,814,602–47,845,820, comprised 16 exons, 15 of which are coding. Eighteen amplicons were generated encompassing all exons and their flanking 5′ and 3′ UTRs and intronic sequence using primers flanking each exon (Appendix 2A), and sequenced. Ten exonic variations were identified (Appendix 4B). Two (Nos.1 and 2) were in the 5′ UTR in exons 1 and 2, respectively; five (Nos. 3–7) were in exonic coding sequence but all represented synonymous third-base codon changes and two (No. 9 and 10) were in the 3′ UTR.

Furthermore, for eight of these ten *SAG* variations, all Basenjis (affected and normal dogs) were homozygous for one allele that differed from the allele in the boxer and the reference sequence. The two exceptions were variations 8 and 9, which segregated informatively. Since SNP number 9 was located in the 3′ UTR of the gene, it seemed unlikely to represent a causative mutation.

**Figure 4:**
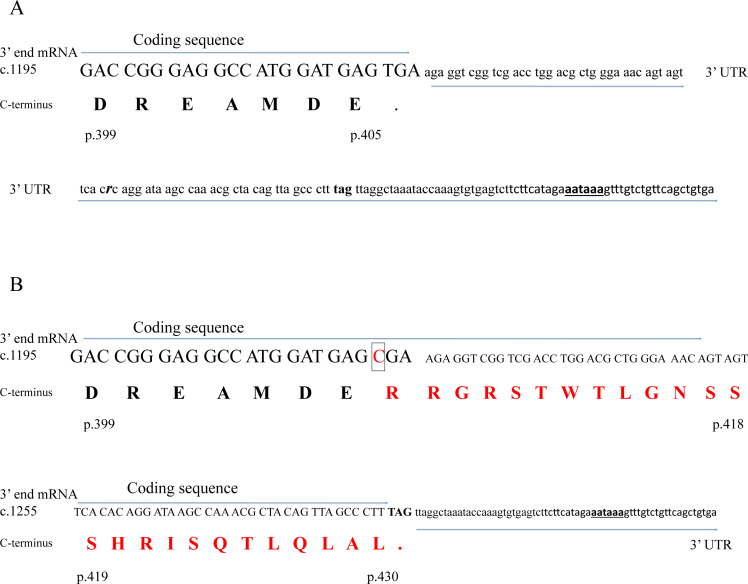
Sequence of the 3′ end of canine S-antigen messenger ribonucleic acid and the deduced translated protein. Uppercase plain letters=coding nucleotide sequence; lower case letters=3′ untranslated region (UTR); Uppercase bolded letters=deduced amino acid translation; Period (.) indicates Stop codon. Bold underlined lowercase nucleotides (**aataaa**)=the polyadenylation signal. The sequence ends where a poly A tail was observed in X98460 messenger RNA (mRNA) deposited in the NCBI database. **A**: Sequence from a normal Basenji dog is presented. The normal stop codon (TGA) is codon 406. A single nucleotide polymorphism (SNP) (***r***=A/G) is observed in the 3′ UTR of normal dogs as indicated in bold italics. **B**: Sequence from a Basenji dog affected with progressive retinal atrophy (PRA) is presented. The red-boxed nucleotide (C) in panel **B** is the mutation that alters the normal stop codon (TGA) to code for arginine (R=CGA). Amino acids in red are the additional ones introduced by the nonstop mutation.

Variation number 8 (Appendix 4B), however, was a much more significant tyrosine to cysteine transition mutation at position CFA25:47,845,680 (c.1216T>C; Figure 4) that changed the normal stop codon to code for the amino acid arginine, which would result in a deduced addition of 25 amino acids (p.*405Rext*25) to the normal 405 amino acid protein (Figure 4), and was thus identified as a strong candidate for the causative mutation.

To further evaluate this variation as the candidate mutation, 80 Basenji dogs were then genotyped with PCR amplification and sequencing of *SAG* exon 18 (Table 3). Eighteen of these dogs had been diagnosed as phenotypically normal at an age considered beyond risk for PRA; 19 had been diagnosed as cases affected with PRA, and six were “suspicious for PRA” (i.e., tentatively diagnosed clinically as likely to develop PRA, based on fundus appearance). Phenotypes for the remaining 37 dogs were unassigned, usually because the dogs were too young for a confident diagnosis of PRA when examined. Among the 18 phenotypically normal Basenjis, 17 genotyped as either homozygous for the wild-type allele at variation number 8 or heterozygous for the presumptive mutant allele; the remaining dog (phenotypically normal at 6.4 years of age) was homozygous for the mutant allele. Given the variation in disease onset in some dogs, this finding was not unexpected. Of the 19 dogs affected with PRA, 17 genotyped homozygous for the mutation, and two dogs were homozygous for the wild-type allele. In the PRA-suspicious group, four dogs were homozygous for the mutant allele, and two were heterozygous for the mutation. Of the 37 dogs with no phenotypic assignment, 16 were homozygous for the wild-type allele, 19 were heterozygous for the mutation, and two were homozygous for the mutant allele. An allele-specific extension test was conducted on 110 dogs from 22 different breeds not known to segregate this disease, and none were found to carry this mutation (Appendix 1).

**Table 3:** Genotype survey in Basenji dogs.

**Phenotype**	**Genotype**			Total
	**T/T**	**C/T**	**C/C**	
Normal	7	10	**1**	**18**
PRA affected	**2**	0	17	**19**
PRA suspicious	0	**2**	4	**6**
Phenotype is unavailable	16	19	2	**37**
Total	**25**	**31**	**24**	80

Eighty pedigree-registered Basenji dogs were genotyped for a non-stop *SAG* mutation (c.1216T>C). One phenotypically normal dog genotyped homozygous for the mutation; 2 dogs diagnosed clinically as “PRA affected” genotyped homozygous wild-type, and 2 dogs diagnosed clinically as “suspicious of having PRA” genotyped heterozygous.

### Bronzing phenotype and mutation genotype correlation

The *SAG* mutation in human Oguchi disease is also causally associated with a discoloration of the fundus, termed the Mizuo-Nakamura phenomenon. An altered appearance of the tapetal fundus, characterized by a golden-brown discoloration and peripheral mottling, is also seen on ophthalmoscopy in some Basenji dogs, and termed bronzing. The cases and controls in the present association study were deliberately chosen from among dogs free of this bronzing phenotype, whether affected with PRA or not. To evaluate if this bronzing phenomenon might represent a canine homolog of the human Mizuo-Nakamura phenomenon, a set of 17 dogs diagnosed as exhibiting this bronzing, and 26 dogs free of the trait, were genotyped for the canine *SAG* non-stop mutation. No evidence of correlation between the *SAG* genotype and the presence or absence of this bronzing phenotype was observed (data not shown).

## Discussion

The online Mendelian inheritance in the animal database (OMIA) currently lists 614 traits and disorders in dogs, of which 238 are Mendelian. For 164 of those, the causative mutation is known. More pertinently, 24 mutations have currently been reported in 18 genes as causatively associated with retinal diseases in at least 58 dog breeds. Approximately 12 genes result in a PRA phenotype while the rest affect other retinal cells or structures [13]. Progress in understanding canine population structure, genome history, and the opportunities presented by breeds as isolate populations has enhanced research by enabling new approaches for mutation discovery: from candidate gene approach to linkage studies, through linkage disequilibrium, and most recently GWAS. The increasing number of trait-causative gene discoveries in recent years is testimony to the power of these technologies and approaches, especially when combined appropriately with canine population structure.

Important criteria in designing an association study include accurate phenotypic ascertainment to ensure that cases and controls are assigned correctly, and that all affected dogs share the same disease; avoidance of stratification between cases and controls to prevent differences in population structure between these groups; selection of cases that are the least related to each other, to reduce the LD interval as much as possible; and analysis of pedigrees for evidence supporting inheritance IBD. Since within breeds dogs are relatively genetically homogenous, like isolated human populations, the number of SNP genotypes sufficient for association mapping is estimated to be much lower than in diverse human populations, and the number of cases and controls could be as low as 20 dogs per group [32]. However, the number of dogs required for association mapping is also breed dependent, since variation in genomic structure is evident between breeds, depending on their origin and history, with factors such as bottlenecks impacting haplotypes and heterogeneity. The Basenji breed, although one of the most ancient canine breeds, has low genomic diversity, heterozygosity, and number of haplotypes [32]. This is not surprising since nearly all the Basenjis in the Western world descend from a few dogs originally imported from Africa. This suggested that even lower numbers of cases and controls than usual might be sufficient for an association study in the Basenji compared to other breeds.

In designing the present study, careful selection of cases and controls that most stringently met the GWAS criteria listed was more critical than the number of cases and controls enrolled. In particular, cases were selected that had the most clear-cut and consistent disease (stage II disease), and from pedigree analysis demonstrated probable IBD recessive inheritance; and controls were selected that were free of retinal disease at an age likely to be beyond the apparent age of risk (at least 6 years of age) and were appropriately related to the dogs selected as cases. This reduced the number of “high confidence dogs” selected to just six affected dogs and three controls.

GWAS initially identified three potentially significant loci associated with the disease phenotype, on CFA4, CFA13, and CFA25. Haplotype analysis, assuming recessive inheritance, excluded the CFA4 locus. Although homozygosity blocks were observed on CFA13 and CFA25 in affected dogs, the LD expected from the Basenji breed structure strongly favored CFA25 over CFA13. The success of association mapping combined with homozygosity analysis to map the disease to a single locus using fewer than ten dogs confirms that the minimum number of dogs needed for GWAS is breed dependent, and can be low in breeds where LD blocks are large. However, the extensive LD (in this case 2.09 Mb) presents a potentially frustrating tradeoff. If either no obviously appealing candidate genes had been present within the homozygosity block, or many were, a much larger number of affected dogs would have been needed to reduce the LD, the number of potential causative genes, and the genomic region to be sequenced and further evaluated.

A parallel approach using homozygosity analysis to directly identify candidate regions was also undertaken. This method has successfully mapped autosomal recessive diseases in humans, especially in consanguineous families. Examples include mapping of a severe autosomal recessive RP to subsequently identify a causative mutation in *MERTK* [33]; mapping of an autosomal recessive early onset generalized dystonia to subsequently identify a causative mutation in *THAP1* [34]; and homozygosity mapping in one affected member of a consanguineous family segregating an autosomal recessive RP to identify three candidate regions, totaling 46 Mb, enabling subsequent identification of the causative mutation in *C8orf37* [35]. Because dogs within pedigree-registered breeds comprise relatively homogenous closed populations, the dogs would be expected to have multiple blocks of homozygosity throughout their genome. That said, if dogs affected with an autosomal recessive Mendelian trait are chosen to be closely related enough to ensure IBD inheritance, but not so closely related that homozygosity segments are excessively large or numerous, a homozygosity mapping approach can be successful. In the present study, homozygosity analysis of six affected dogs genotypes reduced the candidate region from the complete genome to just five loci spanning a total of 11 Mb.

**Figure 5:**
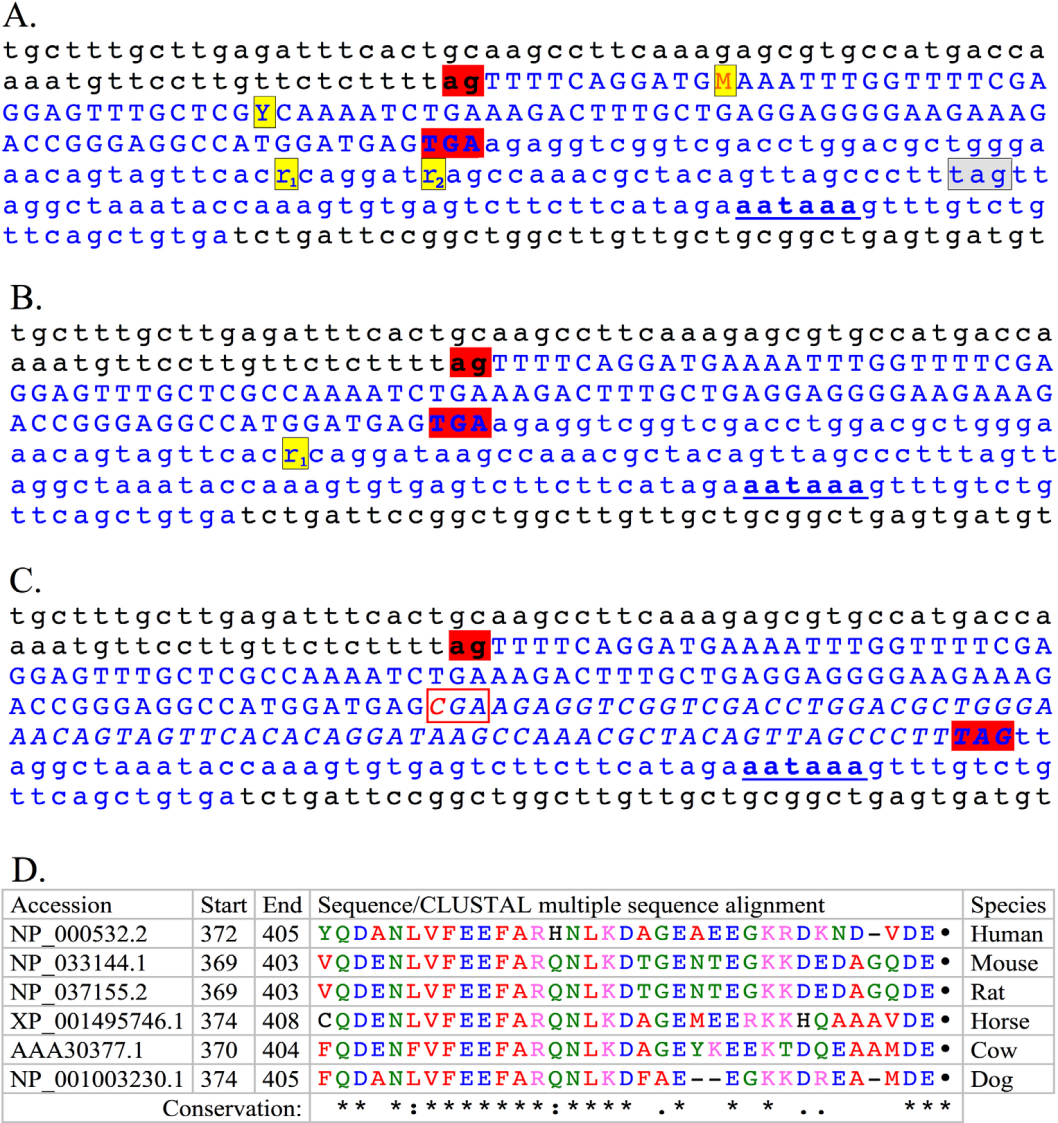
Comparative sequence analysis, 3′ region of canine S-antigen gene. **A**: This panel presents the normal reference canine genomic sequence, from the CanFam3 canine sequence assembly, including the 3' end of intron 15, complete exon 16, and 3′ end of S-antigen (*SAG*). Nucleotides comprising the 3′ end of intron 15, and the genomic sequence beyond the 3′ UTR are in black lowercase. The splice acceptor site, **ag**, at the 5′ junction of intron 15 and exon 16 is highlighted in red and bolded. Blue uppercase characters comprise *SAG* exon 16, the terminal exon, with its stop codon **TGA** highlighted in red and bolded. M=C or A, Y=C or T, indicate observed canine SNPs. Blue lowercase is the 3′ untranslated region (UTR); r1 and r2 are A or G SNPs; boxed in gray is the deduced stop codon in the affected Basenji; bold and underlined is the polyadenylation signal. **B**: The corresponding sequence observed in unaffected Basenji alleles is presented. **C**: The corresponding sequence observed in affected Basenji dogs is presented, in which the stop codon is mutated to a CGA, boxed in red, and the mutation C is labeled in red. The resulting extension of the open reading frame is indicated with italics, and the new deduced stop codon TAG is highlighted in red. **D**: Protein conservation analysis of the amino acids translated from the last exon of *SAG* in six mammalian species. Colors correspond to physicochemical properties of the residues: red=small, hydrophobic and/or aromatic; blue=acidic; magenta=basic; green=hydroxyl, sulfhydryl, or amine; black dot=stop codon. Conservation symbols indicate: asterisk (*)=positions that have a single, fully conserved residue; colon (:)=conservation between groups of strongly similar properties - scoring >0.5 in the Gonnet PAM 250 matrix; period (.)=conservation between groups of weakly similar properties - scoring=<0.5 in the Gonnet PAM 250 matrix. Modified from multiple sequence alignment generated by Clustal Omega. Nineteen amino acids and the stop codon are highly conserved among human, mouse, rat, horse, cow, and dog.

Subsequent candidate gene evaluation of the CFA25 interval identified by association and homozygosity mapping identified a homozygous non-stop *SAG* mutation in all six dogs affected with PRA in the study set. Non-stop mutations are point mutations that convert the stop codon to code for an amino acid, resulting in a deduced elongation of the C-terminus of the protein since the translation continues into the (normal) 3′ UTR. In one human study, such non-stop mutations represented about 0.2% of the codon-changing mutations (119 examples in 87 genes) [36]. These mutations can potentially affect mRNA fate in terms of stability, localization, translation, and regulation or have ramifications at the protein level in terms of stability, localization, folding, regulation, and protein–protein interaction. In the mutant Basenji *SAG* sequence, the first alternative in-frame stop codon is 72 bases after the mutated stop codon and 37 bp upstream from the polyadenylation signal, suggesting that the mutated protein, if translated, would be extended by a further 25 amino acids (Figure 4, Figure 5A-C). This appears to be a long enough abnormally extended sequence to support causal association with a clinical phenotype [37]. Veske et al. [38] previously amplified a full-length cDNA *SAG* from a normal canine retina. Sequence alignment of this cDNA to the canine genomic CanFam2.0 and CanFam3.0 sequence assemblies identifies GT-AG donor-acceptor sites for all introns, and a stop codon at position 1216–1218 in the last coding sequence. We amplified full-length canine *SAG* retinal cDNA that yielded the same open reading frame as Veske et al. [38] and no other splice variants (data not shown). Moreover, protein conservation analysis shows that the stop codon is highly conserved (Figure 5D), suggesting the significance of termination of the C-tail at this position. Either nonsense-mediated decay or a protein misfolding response would lead to loss of *SAG* function. Alternatively, if the protein is successfully translated, its function would also likely be impaired.

Computational protein analysis of the deduced protein (expasy) indicates that the additional 25 amino acids would change the isoelectric point to 6.78 (from 6.0 in the wild-type) and would add four positively charged residues, for a total of 56 in the mutant versus 52 in the wild-type. Although the estimated half-life, predicted localization, and instability index would not change, it is not certain that the mutant protein would fold correctly. If it does, then the mutant protein's ability to bind appropriately to its G-protein-coupled receptor, rhodopsin, would likely be impaired, and this would likely lead to the retinal degeneration phenotype. *SAG* (arrestin) is critically involved in quenching the photoactivated phosphorylated rhodopsin apoprotein (P-Rh*), a process that involves conformational changes in rhodopsin and *SAG* [39,40]. Crystal structure studies have shown that arrestins are elongated two-domain molecules, with their C-tail anchored to the body of their N-domain by bulky hydrophobic residues, which stabilizes their basal (inactive) conformation [41-44]. Receptor binding induces the release of the C-tail and movement of the two domains relative to each other [39,40]. If the mutant *SAG* protein were produced, an elongation of its C-tail by 25 amino acids would likely impair its ability to be anchored in its N-domain and to respond accurately, appropriately and in a timely manner to changes in its receptor.

Because the *SAG* gene product serves to turn off activated rhodopsin during the phototransduction cascade, it may be that the “toxic molecule” in *SAG* associated retinal disease is the activated but unquenched opsin apoprotein. This has similarly been suggested as the pathogenetic mechanism in the T4R opsin mutant dog [45,46] and the T17M opsin mutant mouse [47], in both of which light exposure dramatically accelerates the rate of retinal degeneration. Similarly, in the retinas of *SAG* knockout mice, photoreceptors are progressively lost when the mice are maintained in cyclic light, photoreceptor degeneration was prevented by dark-rearing the mice, and the degeneration was markedly accelerated when the mice were exposed to constant light [48]. No evidence of such light-sensitivity affecting the course of retinal disease in Basenjis has yet been adduced, but this issue needs to be further evaluated.

In human populations, *SAG* mutations have previously been associated with Oguchi disease [10,27,29,49-52] and RP [10,11,28]. In some families, different individuals homozygous for the same mutation can be affected either by Oguchi disease, RP, or both [10,11]. The exact mechanism for such a wide range of clinical expression is unknown, although variation in light exposure has been proposed as a possible modifying factor, based largely on the studies in mouse models [48]. We found no significant correlation between bronzing of the fundus and the SAG nonstop mutation. This suggests that other environmental or genetic factors likely are responsible for this bronzing phenomena in the retina of Basenji dogs and that the bronzing phenomenon does not constitute a risk factor for a Basenji developing this form of PRA.

In screening a larger set of 43 ophthalmologically evaluated Basenji dogs in the present study, two dogs diagnosed clinically as affected with PRA were identified as homozygous for the *SAG* wild-type allele; another 6.5-year-old dog diagnosed clinically as nonaffected, and initially considered to be beyond the risk age, was identified as homozygous for the *SAG* mutant allele; and two dogs that had been diagnosed as “suspicious for PRA” at 8 years of age and older were genotyped as carriers for the mutation (Table 3). As in other breeds [14,53,54], it is likely that more than one PRA-causing mutation is segregating in the Basenji breed, and significant modifying influences—either genetic or environmental—also influence the clinical expression of disease caused by the primary mutation. Human patients with the same *SAG* mutation as each other can also present a broad spectrum of phenotypic expression, and this variation is seen even within a single family, with one member affected with the Oguchi phenotype and another with RP [11]. Clinical manifestations associated with an arrestin 1147delA mutation can range from Oguchi disease and Oguchi disease with partial chorioretinal degeneration to RP with or without a golden yellow fundus reflex [55].

Within the 2.09 Mb LD-homozygous interval identified as the disease locus, four refseq genes are located (*EIF4E2*, *SAG*, *UGT1A6*, *TRPM8*) and more than ten non-refseq genes. Because the present studies were undertaken in a patient population for which only phenotypic data and DNA were available, ongoing and future studies in purpose-bred dogs will provide tissues, previously and currently unavailable, for functional, molecular, transcriptomic, proteomic, and cytologic studies to address critical questions that arise from the present investigations, particularly the effect of the non-stop mutation on the pathology of the disease, and the involvement of other genes within the LD interval on the course of the disease. Basenji dogs affected with PRA can thus serve as a novel large animal model of *SAG*-associated retinal degeneration, offering investigational opportunities to better understand the primary disease mechanism, explore causes for the wide variation of disease as observed in humans and Basenji dogs, investigate the effect of the non-stop mutation on residual function of the protein, test the potential role of light-exposure on the phenotype, and evaluate potential therapeutic approaches.

## Appendix 1. Breeds and number of dogs per breed screened for *SAG* mutation.

To access the data, click or select the words “Appendix 1.” A total of 110 dogs from 22 different breeds were tested for *SAG* mutation. None of these dogs carry the mutant allele.

## Appendix 2. Primers used to screen candidate genes within the LD interval on CFA25.

To access the data, click or select the words “Appendix 2.” **A**. Primer information used for *SAG* screening. **B**. Primer information used for *KCNJ13* screening. **C**. Primer information used for the screening of the non-stop mutation in SAG. In bold and lower case is the base before the last that was altered from the database in C1 and C2 forward primers. In red is the mutated base.

## Appendix 3. Genotype calls for the significant locus on CFA25.

To access the data, click or select the words “Appendix 3.” Allele are represented as A or B. Colored in green are homozygous genotypes for the A allele; Colored in orange are homozygous genotypes for the B allele; Colored in yellow are the heterozygous genotypes AB. NA=call is not available. SNPs within the SAG gene are marked in red. SNPs with the highest -log10(P) values are highlighted in purple. The homozygous block is boxed. The beginning and end of the homozygous block identified by homozygousity analysis are bold.

## Appendix 4. Exonic polymorphisms (SNPs) identified within 2 genes (*KCNJ13* and *SAG*) evaluated as positional candidates for Progressive Retinal Atrophy (PRA) in Basenji dogs.

To access the data, click or select the words “Appendix 4.” All exons from both genes were amplified and sequenced from a Boxer, two unaffected Basenjis, one PRA affected Basenji and its unaffected sibling. **A.** Only one exonic SNP was identified in *KCNJ13*; this was not in association with the Basenji PRA phenotype. **B.** Ten exonic SNPs were identified in *SAG*. Two (Nos.1 and 2) were in the 5′ UTR of exons 1 and 2, respectively; 5 (Nos. 3–7) were in exonic coding sequence but each one was a synonymous third-base codon change; and two (Nos. Nine and 10) were in the 3′UTR. SNP No. 8 (in red), a T>C transition at position 47,845,680 changes the normal stop codon to code for arginine, and was thus identified as a candidate for the causative mutation. In bold are coding sequence SNPs.
